# Numerical Simulation of Continuous Extraction of Li^+^ from High Mg^2+^/Li^+^ Ratio Brines Based on Free Flow Ion Concentration Polarization Microfluidic System

**DOI:** 10.3390/membranes11090697

**Published:** 2021-09-10

**Authors:** Dongxiang Zhang, Xianglei Zhang, Leilei Xing, Zirui Li

**Affiliations:** 1 College of Mechanical and Electrical Engineering, Wenzhou University, Wenzhou 325000, China; dxzhang@stu.wzu.edu.cn; 2 School of Mechanical Engineering, Hebei University of Technology, Tianjin 300401, China; x18716051739@163.com; 3 National Engineering Research Center for Technological Innovation Method and Tool, Tianjin 300401, China

**Keywords:** nanofluidic, lithium extraction, membrane separation, ion concentration polarization

## Abstract

Ion concentration polarization (ICP) is a promising mechanism for concentrating and/or separating charged molecules. This work simulates the extraction of Li^+^ ions in a diluted high Mg^2+^/Li^+^ ratio salt lake brines based on free flow ICP focusing (FF-ICPF). The model solution of diluted brine continuously flows through the system with Li^+^ slightly concentrated and Mg^2+^ significantly removed by ICP driven by external pressure and perpendicular electric field. In a typical case, our results showed that this system could focus Li^+^ concentration by ~1.28 times while decreasing the Mg^2+^/Li^+^ ratio by about 85% (from 40 to 5.85). Although Li^+^ and Mg^2+^ ions are not separated as an end product, which is preferably required by the lithium industry, this method is capable of decreasing the Mg^2+^/Li^+^ ratio significantly and has great potential as a preprocessing technology for lithium extraction from salt lake brines.

## 1. Introduction

Global demand for lithium has increased significantly over the past decades, driven by the expanding requirement of rechargeable lithium batteries for portable electronic devices, electric vehicles, and grid storage applications. Generally, lithium has been obtained from two sources—hard rock ores and continental brines [1,2]. Extraction of lithium from ores/minerals is mainly through calcination. In this method, lithium ore is calcined or roasted and then leached to dissolve lithium into the liquid phase. In this process, a large amount of hydrogen chloride gas is produced, which has serious pollution consequences [3]. As a result, lithium production from alternative resources, i.e., salt lake brines, is increasing steadily over the last decades [4].

Essentially, the key task of lithium extraction from salt lake brines is the separation of Li^+^ and Mg^2+^ ions. This is because magnesium and lithium elements possess similar chemical properties, and magnesium must be removed in most lithium applications [5,6]. So far, several methods have been developed for extracting lithium from salt lake brines, such as precipitation [7,8], adsorption [9], solvent extraction [10,11], calcination [12], and membrane approaches [13,14], etc.

Generally, the precipitation method uses chemical reactions to convert Li^+^ into insoluble substances and then separate them from the solution. This method is commonly used in low Mg^2+^/Li^+^ ratio brines of salt lakes in South America, yielding the largest portion of the world’s lithium productions today [15]. However, this procedure needs a large area and a long processing time [16], and it does not work for high Mg^2+^/Li^+^ ratio salt lake brines. The adsorption method uses ion sieve adsorbents to absorb Li^+^ selectively from the brine and extract lithium in subsequent stages. It works well for high Mg^2+^/Li^+^ ratio brines. Still, its processes are complicated, time-consuming, and suffering from the cost and efficiency problems of the absorbents. Solvent extraction uses an organic extractant to extract Li^+^ from the brine. However, the organic extractant may solve into the brine in this process, causing environmental pollution [17]. Calcination technology uses dried salts obtained from the brines through heating and dissolution. The production procedures and the suffering problems are the same as those in the calcination of mineral ores [18]. Membrane technologies use membranes to separate Li^+^ and Mg^2+^, which could be driven by mechanical pressure (nanofiltration) [13,14] or by the electric field (electrodialysis) [19]. In a nanofiltration system, high pressure is used to push Li^+^ through the nanoporous membranes while Mg^2+^ cannot pass through. The advantages of nanofiltration are low energy consumption, environmental friendliness, and easy operation. However, the total salinity of the feed solution must be low, and the cost of an ion-selective nanofiltration membrane is high [20]. In an electrodialysis system, a monovalent ion exchange membrane is used to transport monovalent Li^+^ through the membrane while blocking the permeation of multivalent Mg^2+^ [21,22]. This method requires high-quality membranes, the cost of which is still hindering its industrial applications. Further research is required to develop new technologies based on completely new fundamental principles. In this aspect, the ion concentration polarization (ICP) based method may play a promising role.

A preferable method for lithium extraction must be able to concentrate Li^+^ and remove Mg^2+^ simultaneously. For the first task, i.e., the concentration of a specific kind of ions, or generally a charged species, Wang et al. developed a micro-nanochannel ICP system to preconcentrate proteins and peptides [23]. In their pioneer system, two microchannels (the main channel and a buffer one) are connected by an array of nanochannels. An electric field is applied through nanochannels from the main channel to the buffer channel. Because the walls of nanochannels are negatively charged, only cations and positively charged species can pass through nanochannels. As a result, the concentration of ions in the main channel near micro-nanochannel interfaces is lower than that in the buffer channel, i.e., ICP is induced across two ends of nanochannels [24,25,26,27]. If the electric field across two microchannels is so high that the concentration of the ions is close to zero near the openings of nanochannels in the main channel, an ion depletion zone (IDZ) is formed there. The electric field in IDZ is significantly higher than those at other locations [28]. This locally amplified electric field in IDZ can hinder the fluid-drag motion of the co-charged species (with respect to the charges on the nanochannel walls). In Wang’s work [23], when the negatively charged proteins or peptides migrate from the inlet to the outlet (carried by fluid flow), they are subjected to a sharply increasing resistive electric force at the boundary of the IDZ. For a specific molecule, if the electric force (**F_E_**) applied to it becomes equal to the fluid drag force (**F_D_**) at a specific location, it will be trapped there. When a large number of molecules arrive at this location, they gather and get concentrated there [29]. Using this method, Wang et al. achieved a million-fold concentration for protein and peptide molecules [23].

If there is more than one type of co-charged species, their force balances (between **F_D_**, proportional to the size, and **F_E_**, proportional to the charge) will be different. As a result, they will be focused at different locations in the microchannel. If one applies a pressure-driven flow to make the fluid drag force **F_D_** of a charged molecule greater than the maximum **F_E_** at the boundary of IDZ, this molecule may *squeeze* through IDZ and flow out of the microchannel. In the meantime, other co-charged molecules remain trapped at the boundary of IDZ because their fluid drag forces are lower than the maximum electric forces at that location. In this way, selective trapping and separation of co-charged species are realized. Based on this understanding, Ouyang et al. selectively focused DNA molecules in the microchannel but let the proteins pass through [30]. Gong et al. studied the applicability of this mechanism for the separation of Li^+^ and Mg^2+^ through numerical simulation. They achieved a very low Mg^2+^/Li^+^ ratio in their product solution, but Li^+^ concentration is also very low [31,32]. Later the same group proposed a method to collect Li^+^ enriched solution selectively through a branch channel, achieving simultaneous concentration of Li^+^ and removal of Mg^2+^ [32].

In recent work, Papadimitridou et al. reported a new system to focus (and separate) charged particles [33]. In their system, sample solutions were pumped horizontally from left to right (as in Figures of [33]) through a macroscale chamber, with all the chamber walls formed by large arrays of microchannels. In the meantime, an electric field was applied in the perpendicular direction (vertically from top to bottom as in Figures of [33]). Nafion membranes are embedded across the microchannel arrays outside the bottom boundary of the chamber to facilitate the cation-selective transport and generate the ICP effect there. The vertical electric field also produces an electroosmotic flow from top to bottom, which carries all the species in the solution to move downwards and facilitates focusing of negatively charged particles while migrating from left to right along with the pressure-driven flow. Based on the fact that the solution runs continuously through the macroscale chamber, with charged species focused (through ICP) and collected at the bottom right corner, this device was referred to as a free flow ICP focusing (FF-ICPF) system. Their FF-ICPF device operates in two modes: peak mode (the concentration of the focused analytes is significantly lower than that of buffer ions) and plateau mode (concentrations of the focused analytes are high enough to neutralize the counterions in the buffer). Separation of different species can be performed in the peak mode by collecting the solutions at different groups of microchannels at the outlet (at the right boundary of the chamber). The FF-ICPF system in [28] can process the sample at a flow rate of 10 µL/min, significantly faster than typical microfluidic systems. However, its enrichment factor is low: ~17 times for three dyes in peak mode and 4–5 times in plateau mode, which is orders lower than the single-channel devices proposed by Wang et al. [23]. In fact, concentrations of focused analytes in the peak mode and the plateau mode had been termed as “electrokinetic limit” and “neutrality limit” by Ouyang et al. in their theoretical work, where approximated analytical expressions and scaling laws were given rigorously [34].

Now that the FF-ICP system has the potential to focus and separate the charged species at a macroscopic scale, one may ask if such a system is capable of concentration and separation of simple ions. This paper will answer this question through numerical simulation. More specifically, we will evaluate the performance of the FF-ICP system in separating Li^+^ and Mg^2+^ ions and check its potential role in lithium extraction from high Mg^2+^/Li^+^ ratio salt lake brines.

## 2. Method

### 2.1. Physical Setup

Similar to the device in [33], the microfluidic device we study is composed of a chamber surrounded by four microchannels arrays. Surfaces of the microchannels at the upper and lower sides of the chamber are positively charged. Under the microchannels at the upper side of the chamber, an anion exchange membrane (AEM) is embedded. Mixed Li^+^, Na^+^, Mg^2+^, K^+^, and Cl^−^ ions flow from left to right, driven by external pressure. 

In the meantime, microchannels at the upper and lower sides of the chamber are connected to reservoirs of NaCl solution. A strong electric field is applied from top to bottom. This electric field will apply an upward **F_E_** to anions and a downward **F_E_** to cations and produce IDZ near the AEM at the interfaces between microchannels and the upper side of the chamber. This electric field also generates an upward electroosmotic flow, which applies an additional component to **F_D_** of all the species in the chamber, on top of the fluid drag imposed by the rightward pressure-driven flow (PDF). As a result, when a cation, e.g., Li^+^ or Mg^2+^, enters the chamber from the left side, it is subjected to an up-right directed **F_D_** and a downward **F_E_**. If the drag force in the *y*-direction *F_D,y_* is greater than the *y*-component of the electric force *F_E,y_*, the ion will move upward when moving rightward with the fluid.

**Figure 1:**
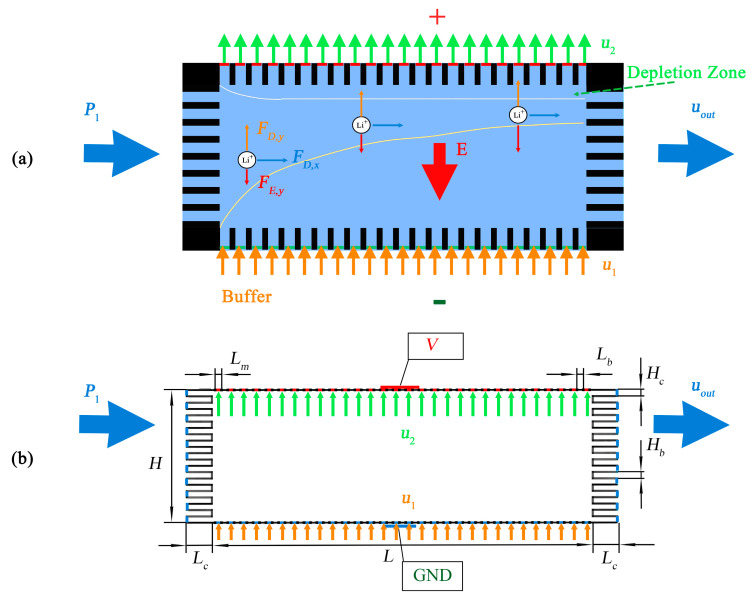
(**a**) Schematic of FF-ICP system for ion concentration and separation. (**b**) The simulation model.

On the other hand, if *F_D,y_* is smaller than *F_D,y_*, the ion moves downward. If the *y*-components of two forces are equal, the ion will be balanced in that location. Because of the ICP effect, the electric field at the boundary of IDZ is significantly higher than in the other regions. These uneven distributions of electric force define different equilibrium positions in *y*-direction for different cation species. As shown in Figure 1a, as Li^+^ ion enters the chamber at the center of the inlet, it is subject to a larger *F_D,y_* than *F_E,y_* (because the downward electric field there is low). Hence, it will move upward as it is moving with fluid in the right direction. When it is closer to the IDZ region, the electric field will become stronger, and the electric force will increase. This upward motion will stop when the two forces (*F_D,y_* and *F_E,y_*) are equal. It will move rightward and run out through microchannels at the right side of the chamber. In the meantime, Mg^2+^ is subject to a 2-times higher electric field and a 1.46-times (calculated from the diffusion coefficients shown in Table 1) higher fluid drag force as compared to those of Li^+^, the balance position for Mg^2+^ will be lower than that of Li^+^. If we optimize the fluid flow speeds and the electric field such that the focused peaks of Li^+^ and Mg^2+^ run out of the system through a different group of microchannels, these two ion species are separated.

### 2.2. Simulation Model

We use a simplified model for our simulation study, as shown in Figure 1b. In this model, the chamber is of length *L* and width *H*. At the left and right sides of the chambers, there are a number of microchannels of length *L_c_* and width *H_c_*. On the upper and lower boundaries of the chamber, there are a series of barriers of length *L_b_* (representing the barrier between microchannels in Figure 1a), the distance between, which is *L_m_* (representing the microchannels). We apply a constant inlet flow of *u*_1_ and a constant outlet flow of speed *u*_2_ at the location of the microchannels on the lower and upper boundaries, respectively. This treatment is based on the fact that the electroosmotic flow has the plug shape in a microchannel, and the concentration of the solution in the lower and upper sides is different. At the inlets of microchannels at the left side of the chamber, constant pressure of *P_1_* is applied. At the outlet of microchannels on the right side, a constant flow speed *u_out_* is set. 

To simplify our computation model, we move the AEM embedded to the microchannels at the upper side of the chamber to the chamber boundary (the green sections). For the electric field, a positive electric potential *V* is applied on these membranes’ segments. At the lower boundary of the chamber, the segments corresponding to the microchannels are grounded. 

### 2.3. Governing Equations

For the fluid flow, the Navier–Stokes equation for an incompressible Newton fluid is employed.

(1) $$\rho \left(\frac{\partial U}{\partial t}+(U\cdot \nabla )U\right)=-\nabla P+\eta \nabla^{2}U-\rho_{e}\nabla \Phi$$

(2) ρ∇⋅U=0

Here ρ is the fluid mass density, U is the fluid velocity, P is the pressure, and Φ is electric potential. 

Transport of charged species is modeled via the Nernst–Planck equations and the mass conservation equation.

(3) $$J_{i}=-D_{i}\nabla C_{i}-Z_{i}\left(D_{i}F/RT\right)C_{i}\nabla \Phi +UC_{i}$$

(4) $$\nabla \cdot J_{i}=-\frac{\partial C_{i}}{\partial t}$$

where $J_{i}$, $C_{i}$ and $Z_{i}$ are flux, concentration, and valence, all for species i. For convenience, we set *i* = 1 for Li^+^, *i* = 2 for Na^+^, and *i* = 3, 4, 5 for Mg^2+^, K^+^, and Cl^−^, respectively. Symbols F, R, and the parameter T are the Faraday constant, gas constant, and temperature, respectively. The Poisson equation governed the distribution of the electric field.

(5) $$-\nabla \cdot (\varepsilon \nabla \Phi )=\rho_{e}$$

where $\rho_{e}=e\sum Z_{i}C_{i}$ is the charge density, with *e* representing the elementary charge and ε denoting the dielectric permittivity of the solution.

### 2.4. Boundary Conditions

As shown in Figure 1b, the boundary conditions illustrate as follows:

On the inlet boundaries of microchannels at the left side, (i) the pressure is *P*_1_; (ii) the concentration of all ionic species is equal to those in the reservoir:

(6) $$P=P_{1},\;C_{i}=C_{i,0},\;i=1,\;2,\;\ldots ,\;5$$

On the outlet boundaries of microchannels at the right side, (i) the free boundary condition is applied for mass transport; (ii) the fluid flow speed is the constant $u_{\mathrm{out}}=(u_{\mathrm{out}},0)$:

(7) $$U=u_{\mathrm{out}},\;\nabla C_{i}\cdot n=0,\;i=1,\;2,\;\ldots ,\;5$$

where n represents the outer-pointing normal vector perpendicular to the boundary of the fluid domain. 

On the microchannel portion of the lower boundary, (i) the electric potential of the lower boundary is 0; (ii) the velocity of the fluid is constant $u_{1}=(0,u_{1})$; (iii) the concentration of Na^+^ is *C*_2,1_, the concentration of Cl^−^ is the opposite number of the sum of other four cations:

(8) $$\Phi =0,\;U=u_{1},\;C_{2}=C_{2,1},\;C_{5}=-\sum C_{i}Z_{i},\;i=1,\;2,\;3,\;4$$

where $c_{i}$ and $Z_{i}$ represent the variable concentration and the valence of ion *i*. 

On the red dashed boundary, (i) a voltage *V* is applied on the membrane to generate electric field; (ii) the velocity of the fluid is the constant velocity ($u_{2}=(0,u_{2})$); (iii) the concentration of Cl^−^ at the upper boundary is $C_{m}$; (iv) fluxes of cations across the membrane are zero. The corresponding equations can be expressed as:

(9) $$\Phi =V,\;U=u_{2},\;C_{5}=C_{m},\;J_{i}\cdot n=0,\;i=1,\;2,\;3,\;4$$

The other black lines and black dashed lines are the microchannel walls, (i) no-slip condition for fluid velocity; (ii) impermeability to all anions and cations:

(10) $$U=0,\;J_{i}\cdot n=0,\;i=1,\;2,\;\ldots ,\;5$$

### 2.5. Numerical Methods

Simulations were carried out using COMSOL Multiphysics software (version 5.6) on a Dell workstation (Precision 7920) equipped with an Intel Xeon processor (Gold 6128) and 112 GB of RAM. Steady-state simulations were used for all studies. Solution convection was modeled with the “Creeping Flow” interface. Moreover, the “Transport of Diluted Species” and the “Electrostatics” interfaces were coupled to solve the “Nernst–Planck–Poisson” equation; 3,833,759 quadrilateral elements were utilized for meshing. Near the membrane region, extremely fine meshes are used to ensure sufficient solution accuracy. To obtain the highly nonlinear solution under high electric potential, we needed to start from low voltage and sweep the high voltage parameter. Initially, the down boundary should be set as no flux for Li^+^, Mg^2+^, and K^+^ to converge the simulation. After the system is stable, we set it out to facilitate ion exchange between the chamber and the reservoir below.

As shown in Figure 1b, in the simulation model, we kept the horizontal microfins to analyze the average ion fluxes of the outflow because the value taken on the boundary will greatly affect the calculation result. However, we ignore the vertical ones as they do not affect the results. 

To analyze the behavior of the separation system, we started with the setting of a particular parameter. Then, we studied the effects of two critical operational parameters, the voltage *V* and velocity **u**, and clarified how these parameters affected system performance to prove the feasibility of the proposed ion separation method. 

## 3. Results and Discussions

In this simulation, the geometric parameters of the chamber are *L* = 90 μm, *H* = 31.5 μm (see Figure 1b). The length of the microfins channel is *L_c_* = 3 μm, and the width is *H_c_* = 1.5 μm, while the distance between the channels is *H_b_* = 1.5 μm. The length of membrane segments is *L_m_* = 1.5 μm, *L_b_* = 1.5 μm.

Using a simplified brine consisting of only five ions (Li^+^, Na^+^, K^+^, Mg^2+^, Cl^−^) [31]. After diluting the raw brine, we selected the following typical concentrations. The concentrations of ions (the left inlet boundary) are: $C_{1,0}=0.001\;\mathrm{mM}$, $C_{2,0}=0.125\;\mathrm{mM}$, $C_{3,0}=0.04\;\mathrm{mM}$, $C_{4,0}=0.04\;\mathrm{mM}$, $C_{5,0}=0.211\;\mathrm{mM}$. The ion concentrations of the lower edge are $C_{2,1}$= 0.375 mM, $C_{5,1}=-\sum C_{i}Z_{i},\;i=1,\;2,\;3,\;4$, where NaCl enters the chamber from the lower boundary as a supplement buffer to maintain electrical neutrality. In the ideal simplified model of the ion-selective membrane, the results of fixed voltage and fixed counter ion concentration are accurate in most cases, especially in the case of high voltage and/or high charge density [36]. At the membrane boundary, the voltage *V* is from *V* = 0 to *V* = 30V_T_, with the thermal voltage V_T_ equal to 25.8 mV [31]. The assumption of charge selectivity is a fixed counterion boundary. We set the fixed concentration of Cl^−^ at $C_{m}=10C_{5,0}$ [37]. As for other parameters, we set *P*_1_ = 100 Pa, *T* = 300 K, ρ = 1000 kg/m^3^, and η = 0.001 Pa⋅s, respectively.

The concentration of Na^+^ is not an essential research item. Although the content of this ion in the brine is high, the Li^+^ can be collected in the subsequent steps [31]. Therefore, it is easy to separate Li^+^ ions from it. Instead, we have to focus on the extraction quantity of Li^+^ ions, especially the Mg^2+^/Li^+^ flux ratio in the microchannel and use this parameter as a separate indicator. Steady-state solutions are used to study the performance of the system. The governing Equations (1)–(5) are solved with the above boundary conditions (6)–(10).

We simulate the steady-state behavior of this system at *V* = 30V_T_, and the PDF constrains the fluid velocity $u_{\mathrm{out}}$ = 0.5 mm/s in the x-direction, and the velocity rate of y-direction is $u_{1}$ = 0.63 mm/s and $u_{2}$ = 1 mm/s.

**Figure 2:**
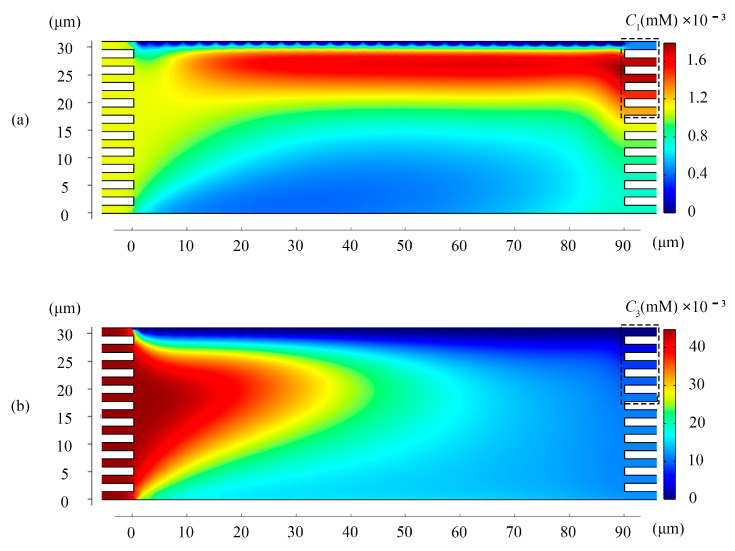
(**a**) The concentration distribution of Li^+^ in the chamber (**b**) The concentration distribution of Mg^2+^ in the chamber. Values of other parameters: *V* = 30V_T_, *u_out_* = 0.5 mm/s, *u*_1_ = 0.63 mm/s, and *u*_2_ = 1 mm/s.

Comparing the two pictures (Figure 2a,b), we can find that in the black dashed box range (18 μm ≤ *y* ≤ 31.5 μm), the Li^+^ ions concentration was much higher than the feed. We put the collector there to get a high-resolution separation effect. The box indicates the location of the collector. 

**Figure 3:**
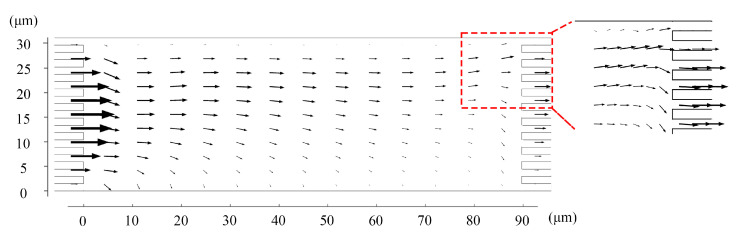
The flux distribution of Li^+^. Values of other parameters are: *V* = 30 V_T_, *u_out_* = 1 mm/s, *u*_1_ = 0.63 mm/s, and *u*_2_ = 1 mm/s.

**Figure 4:**
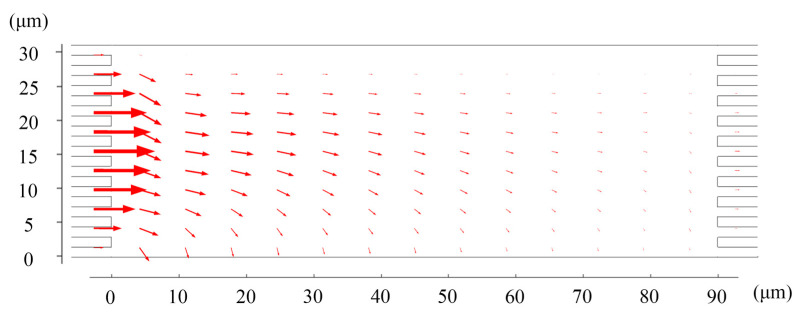
The flux distribution of Mg^2+^. Values of other parameters are: *V* = 30V_T_, *u_out_* = 1 mm/s, *u*_1_ = 0.63 mm/s, and *u*_2_ = 1 mm/s.

Since the initial concentrations were different, we paid more attention to the flux of the two ions. Most Li^+^ flow through the upper right of the chamber where the collector box is located (Figure 3). The flux of Mg^2+^ at the right outlet of the chamber is much lower than the feed (Figure 4). These two figures demonstrated that the proposed system could continuously extract Li^+^ ions with a concentration of 1.28 times in the raw brine while simultaneously removing Mg^2+^ ions.

### 3.1. Effect of the Voltage

**Figure 5:**
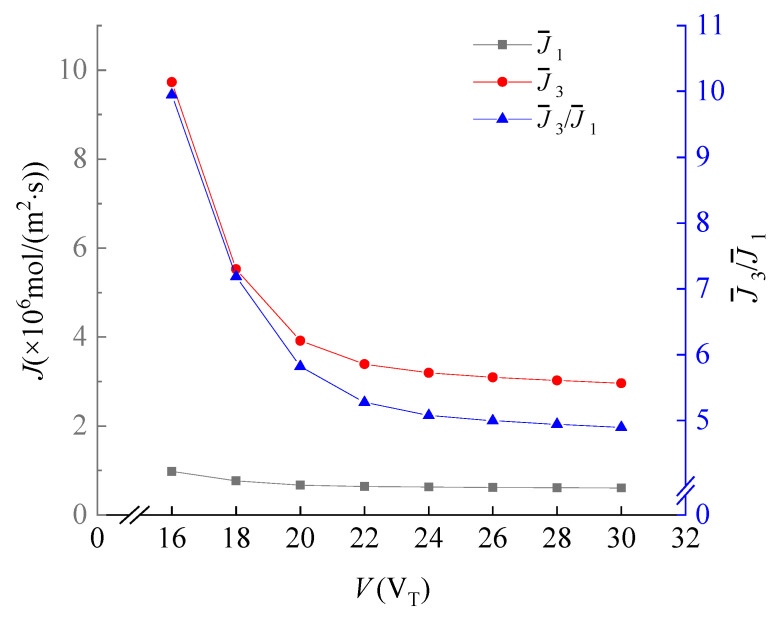
The dependence of the average flux of Li^+^, the average flux of Mg^2+^ and the Mg^2+^/Li^+^ flux ratio on *V*_._ Values of other parameters are: *V* = 30V_T_, *u_out_* = 1 mm/s, *u*_1_ = 0.63 mm/s, *u*_2_ =1 mm/s, 18 μm ≤ *y* ≤ 31.5 μm, *x* = 96 μm.

To reduce the vortices, we control the voltage within 30*V*_T_. It is noticed that only a small fraction of current across the horizontal direction. Hence, the electric field distribution is zero in that direction. When the voltage increases, the IDZ area becomes more extensive, causing the cations to move downwards. Since the **F_E_** of Mg^2+^ ions is significantly greater than other ions, most move to the downside reservoir. The effect of the Mg^2+^/Li^+^ separation is significant before reaching the unstable fluid motion caused by the electric instability (Figure 5) [38,39,40,41,42]. 

### 3.2. Effect of the Velocity u_out_

**Figure 6:**
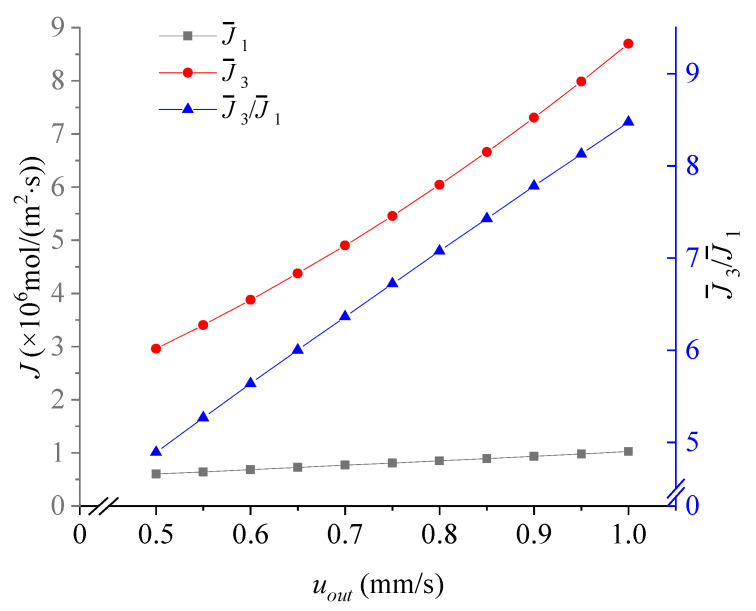
The dependence of the average flux of Li^+^, the average flux of Mg^2+^ and the Mg^2+^/Li^+^ flux ratio on *u_out_*. Values of other parameters are: *V* = 30V_T_, *u*_1_ = 0.63 mm/s, *u*_2_ =1 mm/s, 18 μm ≤ *y* ≤ 31.5 μm, *x* = 96 μm.

As shown in Figure 6**,** when the horizontal speed *u*_out_ increases, the Mg^2+^/Li^+^ flux ratio increased. Although the flux of Li^+^ ions increases, there is not enough time for Mg^2+^ to reach the downside reservoir, resulting in a significant rise in the average flux of Mg^2+^ and poor separation effect, specifically reflected in the increase in the Mg^2+^/Li^+^ flux ratio. 

### 3.3. Effect of the Velocity u_1_

**Figure 7:**
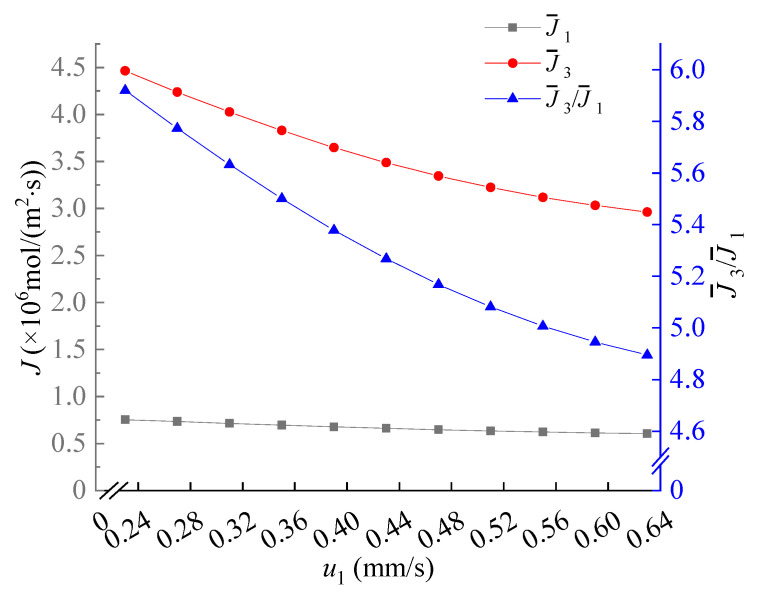
The dependence of the average flux of Li^+^, the average flux of Mg^2+^ and the Mg^2+^/Li^+^ flux ratio on *u*_1_. Values of other parameters are: *V* = 30V_T_, *u_out_* = 0.5 mm/s, *u*_2_ = 1 mm/s, 18 μm ≤ y ≤ 31.5 μm, *x* = 96 μm.

When the speed *u*_1_ increases, as shown in Figure 7, in this case, the separation effect becomes better due to the competition of the co-ions [32]. The Na^+^ supplemented from below increase, the electrophoretic mobility of Na^+^ is between the Li^+^ and Mg^2+^, it would behave as an electrophoretic spacer.

### 3.4. Effect of the Velocity u_2_

**Figure 8:**
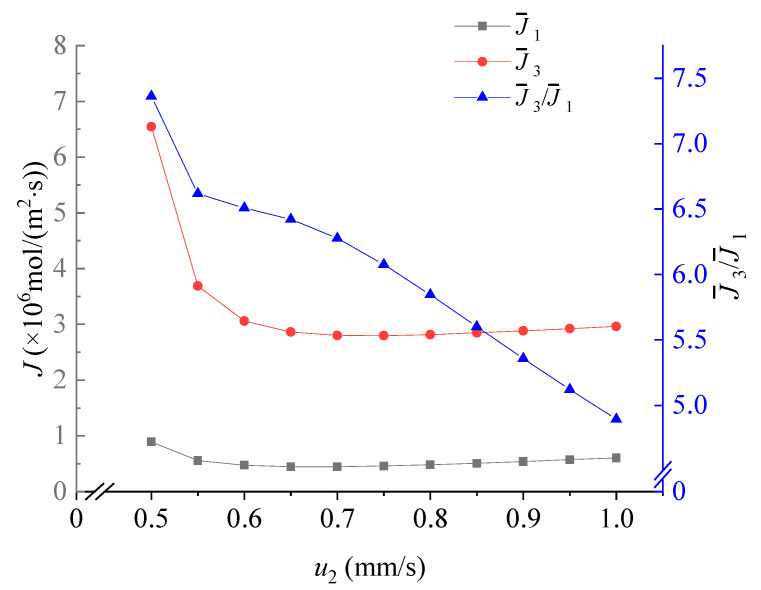
The dependence of the average flux of Li^+^, the average flux of Mg^2+^ and the Mg^2+^/Li^+^ flux ratio on *u*_2_. Values of other parameters are: *V* = 30V_T_, *u_out_* = 0.5 mm/s, *u*_1_ = 0.63 mm/s, 18 μm ≤ *y* ≤ 31.5 μm, *x* = 96 μm.

The velocity *u***_2_** plays a crucial role in this system. As shown in Figure 8, when the *u*_2_ increases, the enrichment effect of Li^+^ ions becomes better. As Li^+^ ions have the lowest electrophoretic mobility, they have the largest upward **F_D_** than the downward **F_E_**. Therefore, they tend to focus near the AEM. Ions with higher valence, i.e., Mg^2+^, are driven to the downside reservoir by the **F_E_**.

### 3.5. Advantages and Limitations

The advantage of this system is that the analytes are continuously flowed through the system by a PDF while vertically separated and concentrated by ICP. Compared to other focusing systems [43], it has a simple structure. More significantly, this method does not involve the chemical reaction of ions in the brine. Compared with other methods such as nanofiltration membrane [14]. Li et al. increased the Li^+^ yield and improved the separation effect of Mg^2+^ and Li^+^ by increasing the operating pressure. The Mg^2+^ rejection rate of 92% was observed at a high-pressure level. Nevertheless, membrane fouling often occurs in nanofiltration separation, and after prolonging the operation time, the separation efficiency is reduced [31]. The typical channel size of our system is the order of tens of microns, so it will not suffer from clogging problems_._ Zhang et al. added new material to the extraction system of polymer inclusion membrane to extract Li^+^ from the Mg^2+^ rich solution (the initial Mg^2+^/Li^+^ molar ratio is 15) [44]. They have increased the extraction rate of Li^+^ by 20%. However, it is necessary to reduce the loss of chemicals additive further to enhance the stability of the membrane. Our system can operate stably without worrying about material loss.

However, some limitations of this work should be noted. First of all, this work is based on first-principles numerical simulation (no experience/parameter approximation). Therefore, the proposed system should be finally verified through experiments. Secondly, in the conventional electro-membrane process, high current density usually produces various nonlinear effects (permeation selectivity loss, heating, over-limit behavior, water splitting effect) [38,45,46,47]. When the electric convection operates in the overcurrent mode, it will form near the ion-selective membrane. The size of these vortices increases with electric potential, which eventually leads to fluid instability. The system can only operate under a certain electric field strength to maintain stability. 

## 4. Conclusions

We have proved the feasibility of continuous separation of Mg^2+^ and Li^+^ ions through numerical simulation based on FF-ICPF. This method could perform enrichment perpendicular to the flow direction of the analyte. Furthermore, the maximum Mg^2+^/Li^+^ ratio can be decreased by ~85% under the electroneutrality limit. However, after trying various channel structures and parameters, the enrichment factor cannot be increased, which is the inherent disadvantage of this system. Numerical simulation shows that this limiting behavior is confined by the accumulation of the charged particles, which affects the shape of the electric field gradient. In the future, if the Joule heating is added, the simulation would be more accurate.

## Figures and Tables

**Table 1:** The transport parameters of the ions in the raw brine [35].

Index *i*	Species	Diffusion Coefficient *D_i_* (×10^−9^ m^2^/s)	Electrophoretic Mobility *μ_i_* (×10^−8^ m^2^/V⋅s)
1	Li^+^	1.029	3.98
2	Na^+^	1.334	5.152
3	Mg^2+^	0.706	7.563
4	K^+^	1.957	5.457
5	Cl^−^	2.032	7.853

## Data Availability

The data that support the findings of this study are available from the corresponding author upon reasonable request.
